# Decreased Vacuolar Ca^2+^ Storage and Disrupted Vesicle Trafficking Underlie Alpha-Synuclein-Induced Ca^2+^ Dysregulation in *S. cerevisiae*

**DOI:** 10.3389/fgene.2020.00266

**Published:** 2020-05-08

**Authors:** Geert Callewaert, Petra D’hooge, Tien-Yang Ma, Mara Del Vecchio, Vincent Van Eyck, Vanessa Franssens, Joris Winderickx

**Affiliations:** ^1^The Yeast Hub Lab, KU Leuven, Kortrijk, Belgium; ^2^Functional Biology, Department of Biology, KU Leuven, Heverlee, Belgium

**Keywords:** yeast, *Saccharomyces cerevisiae*, α-synuclein, Ca^2+^ signaling, vesicle trafficking, pore formation, Ca^2+^ ATPase Pmc1

## Abstract

The yeast *Saccharomyces cerevisiae* is a powerful model to study the molecular mechanisms underlying α-synuclein (α-syn) cytotoxicity. This is due to the high degree of conservation of cellular processes with higher eukaryotes and the fact that yeast does not endogenously express α-synuclein. In this work, we focused specifically on the interplay between α-syn and intracellular Ca^2+^ homeostasis. Using temperature-sensitive *SEC4* mutants and deletion strains for the vacuolar Ca^2+^ transporters Pmc1 and Vcx1, together with aequorin-based Ca^2+^ recordings, we show that overexpression of α-syn shifts the predominant temporal pattern of organellar Ca^2+^ release from a biphasic to a quasi-monophasic response. Fragmentation and vesiculation of vacuolar membranes in α-syn expressing cells can account for the faster release of vacuolar Ca^2+^. α-Syn further significantly reduced Ca^2+^ storage resulting in increased resting cytosolic Ca^2+^ levels. Overexpression of the vacuolar Ca^2+^ ATPase Pmc1 in wild-type cells prevented the α-syn-induced increase in resting Ca^2+^ and was able to restore growth. We propose that α-syn-induced disruptions in Ca^2+^ signaling might be an important step in initiating cell death.

## Introduction

α-Syn misfolding and aggregation are linked to Parkinson’s disease (PD) (Poewe et al., 2017). Duplication or triplication of the *SNCA* gene that encodes α-syn and several point mutations (E46K, A53T, A30P, G51D, and H50Q) are associated with the formation of cytoplasmic protein inclusions and cytotoxicity (Polymeropoulos et al., 1997; Krüger et al., 1998; Singleton et al., 2003; Chartier-Harlin et al., 2004; Zarranz et al., 2004; Tofaris and Spillantini, 2007; Auluck et al., 2010; Lashuel et al., 2012; Appel-Cresswell et al., 2013; Lesage et al., 2013; Ghiglieri et al., 2018). Based on the current knowledge, oligomers of α-syn play a central role in cytotoxicity by impairing a variety of cellular processes. However, the relative contribution of these processes in initiating cytotoxicity and the precise nature of toxic α-syn forms remain uncertain and studies often yield conflicting results (Peelaerts et al., 2015; Pinotsi et al., 2016).

The yeast *Saccharomyces cerevisiae* is a well-founded model system for studying fundamental cellular processes relevant to higher eukaryotes. The high degree of conservation of protein folding and degradation, Ca^2+^ homeostasis and vesicle trafficking between yeast and higher eukaryotes and the fact that yeast does not express homologs of the human synuclein family allow to exploit yeast as a platform to study the molecular mechanisms underlying α-syn cytotoxicity (Franssens et al., 2010). Analysis of various yeast models for α-syn has shown that overexpression of wild-type or mutant α-syn results in growth inhibition and the formation of cytotoxic intracellular inclusions. Several yeast targets for α-syn-induced toxicity have been identified including vesicular transport (Outeiro and Lindquist, 2003; Cooper et al., 2006) mitochondrial functions (Büttner et al., 2008) proteasomal function (Sharma et al., 2006) and Ca^2+^ homeostasis (Büttner et al., 2013; Rcom-H’cheo-Gauthier et al., 2014) each confirming or supporting data obtained in other eukaryotic models. Hence, the cytotoxicity induced by overexpression or mutations of α-syn appears to reflect a combination of different mechanisms acting together or consecutively. Dissecting out the relative significance of each mechanism remains challenging.

In this work, we focus specifically on the interplay between α-syn and intracellular Ca^2+^ homeostasis. The importance of cytosolic Ca^2+^ homeostasis in neurodegenerative diseases, including PD has been underlined in several studies (Chan et al., 2009; Hettiarachchi et al., 2009; Pasternak et al., 2012; Tosatto et al., 2012; Rcom-H’cheo-Gauthier et al., 2014; Angelova et al., 2016). In all eukaryotes, intracellular Ca^2+^ signaling is crucial for multiple biological processes involving channels, co-transporters and pumps. Dopaminergic neurons in PD are particularly vulnerable to disruptions in Ca^2+^ homeostasis due to their distinctive pacemaker activity, which is heavily reliant on Ca^2+^ entry (Pacelli et al., 2015). α-Syn probably interacts with components of the Ca^2+^ toolkit altering Ca^2+^ homeostasis and triggering downstream toxic effects. Previous studies in mammalian cells have mainly focused on the effects of externally applied α-syn oligomers on Ca^2+^ homeostasis. The results of these studies suggest that α-syn oligomers may alter ion (Ca^2+^) homeostasis by either forming membrane pores (leak channels) (Danzer et al., 2007; Angelova et al., 2016) or affecting ion transporters (Ca^2+^ channels and Na^+^/K^+^ pumps) (Adamczyk and Strosznajder, 2006; Shrivastava et al., 2015). Previously, we have shown that expression of α-syn in yeast affects Ca^2+^ homeostasis by increasing basal cytosolic Ca^2+^ levels and TECC responses (Transient Elevation of Cytosolic Calcium) (Büttner et al., 2013). It was demonstrated that chelating free Ca^2+^ with BAPTA or deletion of *PMR1*, encoding a Secretory Pathway Calcium ATPase (SPCA) that transports Ca^2+^ and Mn^2+^ into the Golgi complex, largely suppressed α-syn-induced Ca^2+^ changes. Since Ca^2+^ efflux across the plasma membrane in yeast may rely on vesicular exocytosis, we proposed that defects in vesicular trafficking may underlie α-syn-induced Ca^2+^ dysregulation.

Several studies have demonstrated that α-syn overexpression strongly inhibits vesicle trafficking, thereby disrupting normal processing and transport of proteins within the secretory pathway. At physiological concentrations, the function of α-syn is closely related to SNARE complex formation and/or stabilization (Burre et al., 2014). Whereas α-syn overexpression and the formation of oligomers may inhibit SNARE and RAB/Sec functions leading to reduced secretory activity (Choi et al., 2013). In yeast, it has been shown that several Sec proteins mislocalize to α-syn inclusions while their overexpression reduces α-syn-mediated cytotoxicity (Gitler et al., 2008).

To further unravel the plausible link between α-syn overexpression, Ca^2+^ homeostasis and vesicular transport, we analyzed the effects of α-syn overexpression on intracellular Ca^2+^ handling and storage in temperature-sensitive *SEC4* mutants and deletion strains for the vacuolar Ca^2+^ transporters Pmc1 and Vcx1. We provide evidence that α-syn overexpression in yeast significantly affects basal Ca^2+^ levels and Ca^2+^ storage. Furthermore, we show that α-syn alters Ca^2+^ handling through two distinct mechanisms involving α-syn-mediated disruption of vesicle trafficking and vacuolar Ca^2+^ storage.

## Materials and Methods

### Yeast Strains, Plasmids and Media

Single and double deletion strains were derived from the BY4741 strain background (genotype MATa *his3-1*, *leu20*, *met150*, *ura30*, Euroscarf, Frankfurt, Germany). The *sec4*^*ts*^ strain (strain LRB932, genotype MATa *his3*, *leu2*, *ura3-52*, *sec4-2*, doi: 10.1242/jcs.00203) was derived from the LRB906 strain background (Genotype Mata *his3*, *leu2*, *ura3-52*, doi: 10.1242/jcs.00203). *PMC1* and *VCX1* deletion strains in the BY4741 background were obtained from the yeast deletion collection (Giaever and Nislow, 2014). YPD medium containing 2% peptone, 1% yeast extract and 2% glucose was used for growth and maintenance of yeast cells. Synthetic complete medium containing 0.19% yeast nitrogen base without amino acids, 0.5% ammonium sulfate supplemented with synthetic drop out amino-acid/nucleotide mixture and 2% glucose was used for selection, growth, and maintenance of transformed yeast strains. Transformation of yeast cells was performed following the lithium/polyethylene glycol method (Gietz Daniel et al., 1995). To monitor cytosolic Ca^2+^ levels, strains were transformed with pYX212 vector encoding cytosolic apoaequorin (pYX212-cytAEQ) (a kind gift from E. Martegani, Department of Biotechnology and Biosciences, University of Milano-Bicocca, Milan, Italy) (Tisi et al., 2002). The expression of wild-type α-syn was under the control of the *TPI* promoter (Liu et al., 2012) in the pGGE181 vector (pGGE181-α-syn). Cells transfected with empty vector (EV) (pGGE181-EV) served as controls. To obtain *SEC4* overexpression cells were transformed with pAG423GPD-Sec4 plasmid while the pAG423GPD-ccdB-HA plasmid served as EV control. *PMC1* was expressed with the pYX222 plasmid under control of the *TPI* promoter. For confocal microscopy (Zeiss LSM 710 laser scanning microscope using a 100x high-NA objective) α-syn C-terminally tagged with yeast enhanced green fluorescent protein (α-syn-yeGFP) was expressed in the pYX212 vector (pYX212-α-syn-yeGFP) under control of the *TPI* promoter.

To visualize Pmc1 distribution in α-syn expressing cells, BY4741 cells were co-transformed with pPMC1-GFP fusion plasmid (a kind gift from P.A. Pedersen, Department of Biology, University of Copenhagen, Copenhagen, Denmark) (Scharff-Poulsen and Pedersen, 2013) and pGGE181-α-syn. Cells transformed with pGGE181-EV served as control. For FM4-64 staining of vacuoles, transformed cells were grown in synthetic medium until late exponential phase, incubated with 30 μM FM4-64 (Thermo Fisher) at 30°C for 60 min and washed twice with fresh synthetic medium. Fluorescence was visualized with a Leica DM4000B or DMi8 microscope. Images were deconvolved with Huygens Essential (v18.04, Scientific Volume Imaging) and further processed with Fiji (v1.52p) (Schindelin et al., 2012).

### SDS-Page and Western Blotting

Total protein extracts were prepared from samples taken at 2 OD_600_ (optical density at 600 nm) units in sample buffer containing 50 mM Tris, pH 8, 2% SDS, 0.1% bromophenol blue, 10% glycerol. Antibodies used were: Anti-α-synuclein (Sigma, S3062, 1:1000), Anti-ADH2 (Chemicon, AB1202, 1:10000) and mouse Anti-rabbit complexed with HRP (Santa Cruz, 1:1000). Membranes were blocked in TBS-T (0.05% Tween-20) with 5% BSA. Antibodies were diluted in TBS-T (0.05% Tween-20) with 5% BSA. Primary antibodies were incubated overnight at 4°C. The secondary antibody was incubated for 1 h at room temperature.

### RNA Extraction and qPCR

To determine gene expression, one OD_600_ unit of cells was harvested from overnight cultures grown at the permissive temperature (25°C). Harvested cells were incubated at 37°C for 1 h in growth medium and then switched to 100 μl Y1-buffer (EDTA 0.1 M, Sorbitol 1 M, Lyticase 50 U/ml, 0.1% β-mercaptoethanol, buffered at pH 7.4 with 1 M KOH) for 30 min at 30°C with gentle shaking in order to generate spheroplasts. Total RNA was extracted using the “RNeasy mini kit” (Qiagen). cDNA was generated using the “Transcriptor First Strand cDNA Synthesis Kit” (Roche) starting from 1 μg total RNA, using the Anchored-oligo(dT)_18_ Primers delivered with the kit. Unpurified cDNA was diluted 1/10 before use in quantitative-PCR using the “Lightcycler^®^ 480 SYBR Green I Master” kit (Roche). Primer sets used with respective efficiencies and final concentrations were: (1) Pmc1 forward primer TCACCACGTTTTAGTCGG, Pmc1 reverse primer AGTTATCCACCGGAAATTTCTG, %E = 85, final concentration = 250 nM (2) Act1 forward primer AGGTTGCTGCTTTGGTTATTG, Act1 reverse primer TGACCCATACCGACCATG, %E = 91, final concentration = 250 nM. Program settings were as follows: pre-incubation at 95°C for 10 min; 40 amplification cycles 10 s at 95°C, 15 s at 53°C and 15 s at 72°C. The Pfaffl method was used to determine relative gene expression in order to compensate for varying primer efficiencies. *ACT1* was used as reference gene.

### Cytosolic Ca^2+^ Measurements Using Aequorin

Cytosolic Ca^2+^ levels ([Ca^2+^]_*in*_) were measured in populations of yeast cells expressing apoaequorin as previously described (Büttner et al., 2013). Briefly, yeast cells were transformed with the necessary vectors including pYX212 encoding the apoaequorin gene under the control of *TPI* promoter. Starting from pre-cultures (OD_600_ of ±2–3), two OD_600_ units were plated on Concanavalin A coated coverslips and incubated at 25°C for 1 h. For aequorin reconstitution, cells were washed with 0.1 M 2-(*N*-morpholino) ethanesulfonic acid (MES)/ Tris pH 6.5 and incubated with 0.1 M MES/Tris, pH 6.5 supplemented with 5 μM wild-type coelenterazine (Promega) at either 37°C (non-permissive or restrictive temperature), 30 or 25°C (permissive temperatures) for 1 h. After removing excess of coelenterazine, coverslips with adherent yeast cells were placed in the perfusion chamber of a single-tube luminometer (photomultiplier tube for photon detection (Type H3460-04, Hamamatsu Photonics, Japan) positioned about 2 cm above the coverslip surface) and perfused with the required solutions at either 37°C (non-permissive temperature), 30 or 25°C (permissive temperatures). Adherent cells were initially perfused with 0.1 M MES/Tris, pH 6.5, followed by 0.1 M MES/Tris, pH 6.5 supplemented with 10 mM CaCl_2_. To estimate intracellular Ca^2+^ storage (D’hooge et al., 2015), cells were exposed for 90 s to a Ca^2+^-free medium containing (in mM): 200 KCl, 100 NaCl, 3 EGTA, 20 Hepes/KOH at pH 6.8 and then permeabilized with 0.5% Triton X-100 in the same medium. At the end of all experiments, the residual reconstituted aequorin was completely discharged by perfusing the cells with a solution containing 10 mM CaCl_2_ and 0.5% Triton X-100. Light impulses originating from the entire yeast cell population were discriminated, pre-scaled and integrated (1 s time interval) with a PC-based 32-bit counter/timer board (PCI-6601, National Instruments Corporation, Austin, TX, United States). The emitted light was calibrated offline into cytosolic Ca^2+^ values using the following algorithm [Ca^2+^]_*in*_ = ((L/Lmax)^1/3^+[K_*TR*_(L/L_*max*_)^1/3^-1)/(K_*R*_(L/Lmax)^1/3^]) with K_*TR*_ and K_*R*_ the constants for the Ca^2+^-unbound and Ca^2+^-bound state, respectively (Bonora et al., 2013), L the luminescence intensity at any time point and Lmax the integrated luminescence (Allen and Blinks, 1978; Bonora et al., 2013; D’hooge et al., 2015). Data presented for [Ca^2+^]_*in*_ in this study are all averages of replicate traces (with n the number of coverslips tested) (Supplementary Figure S1).

### Determination of Growth Profiles

Following Ca^2+^ measurements, the same cultures were used to inoculate new cultures at a starting OD_600_ of 0.01. Growth was quantified by measuring the OD_600_ over a 72 h period at 25°C (96-well plate reader Multiskan Go, Thermo Scientific). Absorbance data were averaged for each condition and plotted as a function of time. Based on the log-transformed growth profiles, we calculated the half-times for growth (*t*_50_) of each strain, i.e., the time necessary to reach half-maximal OD_600_.

### Statistical Analysis

Results are expressed as mean ± standard deviation (SD) or standard error of mean (SEM) as indicated. One-way analysis of variance (ANOVA) with Bonferroni posttest and unpaired Student’s *t*-test were used to determine statistical significance between datasets.

## Results

### α-Syn Impairs Ca^2+^ Homeostasis and Inhibits Growth in WT Yeast

**FIGURE 1:**
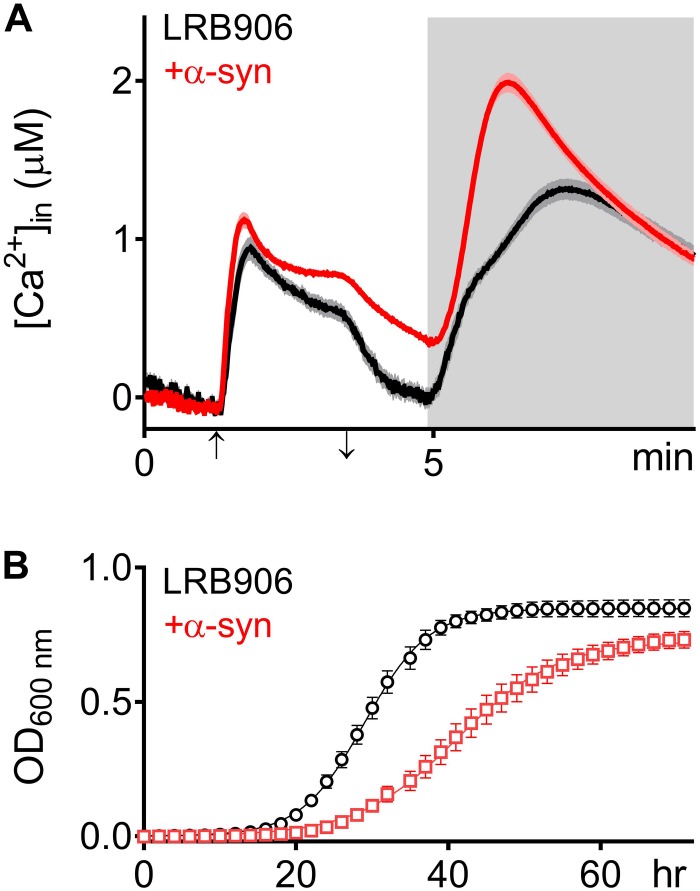
α -Syn overexpression in LRB906 cells. **(A)** Averaged Ca^2+^ transient ± SEM (thin lines either side of the Ca^2+^ transient trajectory) of LRB906 (black trace) and LRB906 cells overexpressing α-syn (+α-syn – red trace) at 37°C. Cells were initially perfused with Ca^2+^-free starvation medium and then transferred to a 10 mM external Ca^2+^ medium for 2 min (indicated by up and down arrow on *X*-axis). Thereafter, cells were briefly exposed to Ca^2+^-free intracellular medium prior to membrane permeabilization using Triton X-100 (indicated by light gray zone). **(B)** Growth curves based on OD_600_ density measurements at 25°C of LRB906 (black) and LRB906 cells overexpressing α-syn (+α-syn – red curve). Each time point represents the average ± SEM of triplicate cultures.

To evaluate the effects of α-syn expression on intracellular Ca^2+^ homeostasis, we monitored cytosolic [Ca^2+^] ([Ca^2+^]_*in*_) after removal of extracellular Ca^2+^ (Rest [Ca^2+^]) and estimated the Ca^2+^ storage by monitoring the release of Ca^2+^ from intracellular stores when cells were permeabilized in Ca^2+^-free medium (Peak [Ca^2+^]). Figure 1A shows the release of stored Ca^2+^ in wild-type LRB906 cells at 37°C (Figure 1A – black trace – see also Supplementary Figures S1A,B). Cells were initially perfused with a 10 mM Ca^2+^ external solution and after switching to Ca^2+^ free medium they were permeabilized using Triton X-100. In 10 mM Ca^2+^ external solution [Ca^2+^]_*in*_ amounted to 530 nM and rapidly decayed in Ca^2+^-free medium to nearly zero. Subsequent permeabilization of the membrane evoked a biphasic Ca^2+^ release transient. The initial fast phase with a time-to-peak of about 50 s, referred to as the ‘fast Ca^2+^ release component’, may correspond to release of free Ca^2+^ stored in organelles, whilst the second larger and slower component with a time-to-peak value of about 2 min 20 s, referred to as the ‘slow Ca^2+^ release component’, may comprise release of Ca^2+^ bound to the organellar matrix and largely reflects vacuolar Ca^2+^ storage (D’hooge et al., 2015). For the LRB906 strains, the [Ca^2+^]_*in*_ values determined at the end of the Ca^2+^-free period before permeabilization (Rest [Ca^2+^]) as well as the peak amplitude of the Ca^2+^ release transient taken as the maximum of the biphasic Ca^2+^ release transient (Peak [Ca^2+^]) are listed in Table 1.

**TABLE 1:** [Ca^2+^]_*in*_ determined at the end of the Ca^2+^-free period (Rest [Ca^2+^]) (before membrane permeabilization) and the peak amplitude of the Ca^2+^ release transient (Peak [Ca^2+^]) in LRB906 yeast strains at 37°C.

3*Rest [Ca^2+^] (μM)	**LRB906** 0 ± 0.20 *n* = 24	***sec4*^*ts*^** 0.12 ± 0.20 *n* = 35	**LRB906-pSEC4** 0 ± 0 *n* = 18
**LRB906+α-syn** 0.29 ± 0.12 *n* = 33	**** <0.0001		
***sec4*^*ts*^+α-syn** 0.53 ± 0.26 *n* = 38		**** <0.0001	
**LRB906-pSEC4+α-syn** 0.15 ± 0.23 *n* = 18			*** 0.0001

3*Peak [Ca^2+^] (μM)	**LRB906** 1.33 ± 0.28 *n* = 24	***sec4*^*ts*^** 3.21 ± 0.66 *n* = 35	**LRB906-pSEC4** 2.0 ± 0.14 *n* = 18

**LRB906+α-syn** 2.07 ± 0.29 *n* = 33	**** <0.0001		
**Sec4^*ts*^+α-syn** 3.71 ± 0.53 *n* = 38		** 0.0024	
**LRB906-pSEC4+α-syn** 2.86 ± 0.63 *n* = 18			**** <0.0001

Data are mean and SD for n cultures (coverslips). Statistical differences were calculated using one-way ANOVA with Bonferroni posttest. **P ≤ 0.01; ***P ≤ 0.001; ****P < 0.0001.

When the same experiment was repeated in LRB906 cells that overexpressed α-syn, cells displayed (1) elevated cytosolic Ca^2+^ levels in 10 mM external Ca^2+^ (775 nM); (2) incomplete decay of [Ca^2+^]_*in*_ after switching to Ca^2+^-free medium (Rest [Ca^2+^] 0.29 ± 0.12 μM); (3) a quasi-monophasic Ca^2+^ release transient with increased peak [Ca^2+^] (Peak [Ca^2+^] 2.07 ± 0.29 μM attained 1 min 15 s after membrane permeabilization) and (4) a faster decay rate (Figure 1A – red trace – see also Supplementary Figures S1C,D).

As shown in Figure 1B, α-syn-induced Ca^2+^ changes were accompanied by significant growth inhibition measured at 25°C. Specifically, LRB906 cells overexpressing α-syn reached half time for growth (*t*_50_) after 42.27 ± 5.84 h, resulting in a significant Δ*t*_50_ of 12.3 h relative to wild-type LRB906 cells transformed with an empty plasmid (*t*_50_ of 29.97 ± 2.99 h). The t_50_ values are listed in Table 2.

**TABLE 2:** Growth of LRB906 yeast strains.

**Growth *t*_50_ (hr)**	**LRB906** 29.97 ± 2.99 *n* = 10	***sec4*^*ts*^** 42.50 ± 1.44 *n* = 11	**LRB906-pSEC4** 27.19 = 1.50 *n* = 9
**LRB906+α-syn** 42.27 ± 5.84 *n* = 16	**** <0.0001		
***sec4*^*ts*^+α-syn** 56.63 ± 4.81 *n* = 11		**** <0.0001	
**LRB906-pSEC4+α-syn** 35.16 ± 3.72 *n* = 12			**** <0.0001

Equal number of cells were grown at 25°C. The *t*_50_ values are the mean and SD for at least 9 replicates. Statistical differences were calculated using one-way ANOVA with Bonferroni posttest. ****P < 0.0001.

### α-Syn Induces a Shift From Biphasic to Quasi-Monophasic Ca^2+^ Release

There is compelling evidence that α-syn interferes with exocytotic pathways in both yeast and mammalian cells (Gitler et al., 2008; Choi et al., 2013; Burre et al., 2014). To exploit whether the α-syn-induced Ca^2+^ changes are directly linked to deficits in exocytosis, we designed a series of experiments using yeast cells carrying a temperature-sensitive (ts) mutation in *SEC4* (*sec4*^*ts*^). Sec4, an ortholog of human Rab8, is a small G-protein required for delivery and initial docking of secretory vesicles at the plasma membrane.

**FIGURE 2:**
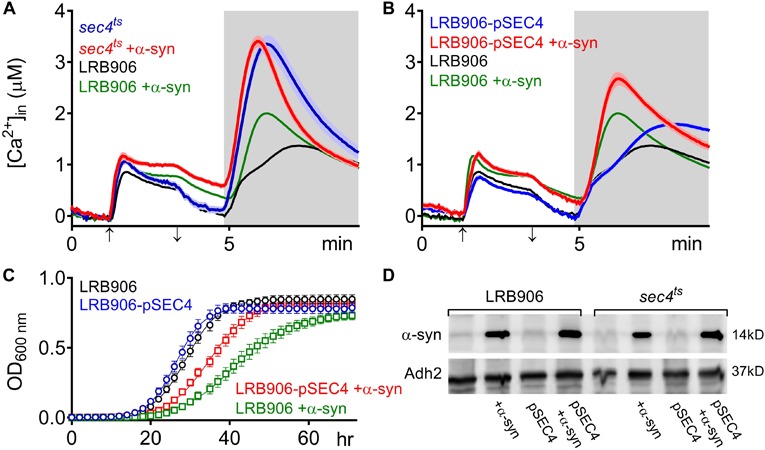
α-Syn overexpression in *SEC4* mutant cells. **(A,B)** Averaged Ca^2+^ transient ± SEM (thin lines either side of the Ca^2+^ transient trajectory) of *sec4*^*ts*^ **(A)** and LRB906 cells overexpressing *SEC4* (LRB906-pSEC4) **(B)** with and without α-syn expression as compared to LRB906 cells at 37°C. Cells were initially perfused with Ca^2+^-free starvation medium and then transferred to a 10 mM external Ca^2+^ medium for 2 min (indicated by up and down arrow on *X*-axis). Thereafter, cells were briefly exposed to Ca^2+^-free intracellular medium prior to membrane permeabilization using Triton X-100 (indicated by light gray zone). **(C)** Growth curves based on OD_600_ density measurements at 25°C. Strains analyzed include LRB906 (black), LRB906 overexpressing *SEC4* (LRB906-pSEC4 – blue), LRB906 overexpressing α-syn (LRB906 +α-syn – green) and LRB906 overexpressing *SEC4* and α-syn (LRB906-pSEC4 +α-syn – red). Each time point represents the average ± SEM of triplicate cultures. **(D)** Western blot of control LRB906 cells and cells overexpressing *SEC4* (pSEC4) with and without α-syn overexpression (first 4 lanes), and control *sec4*^*ts*^ cells and cells overexpressing *SEC4* with and without α-syn overexpression (last 4 lanes). Immunodetection was performed using primary antibodies directed against α-syn or Adh2 as indicated on the left. Molecular weight markers are indicated on the right.

At permissive temperature (25°C), the Ca^2+^ responses in *sec4*^*ts*^ cells are very similar to those in wild-type LRB906 cells showing complete cytosolic [Ca^2+^] decay after switching to Ca^2+^-free medium and biphasic release of stored Ca^2+^ following membrane permeabilization (Supplementary Figure S2). However, as compared to wild-type LRB906 cells, the Ca^2+^ storage in *sec4*^*ts*^ cells was significantly larger (Table 1). In addition, *sec4*^*ts*^ cells grew slower than wild-type LRB906 cells (Table 2). At the restrictive temperature of 37°C, the decay of [Ca^2+^]_*in*_ after switching to Ca^2+^-free medium in *sec4*^*ts*^ cells was somewhat slowed down (Zero [Ca^2+^] 0.12 ± 0.20 μM), but much more strikingly was that the Ca^2+^ release following membrane permeabilization became monophasic with a much higher peak value (Peak [Ca^2+^] 3.21 ± 0.66 μM) and a faster decay rate (Figure 2A – blue trace – see also Supplementary Figure S2) as compared to wild-type LRB906 cells (Figure 2A – black trace). This monophasic Ca^2+^ release transient is clearly reminiscent of the one observed in LRB906 cells overexpressing α-syn (Figure 2A – green trace). Assuming that the large monophasic Ca^2+^ release transient in *sec4*^*ts*^ cells at restrictive temperature reflects a rapid release of Ca^2+^ from clusters or pools of vesicles that can no longer undergo membrane fusion or exocytosis, our results may suggest that the α-syn-induced shift from biphasic to quasi-monophasic Ca^2+^ release reflects the effects of α-syn on vesicular dynamics.

When α-syn was overexpressed in *sec4*^*ts*^ cells (Figure 2A – red trace), we observed that similar to the effects of α-syn in wild-type LRB906 cells, resting [Ca^2+^]_*in*_ in 10 mM external Ca^2+^ was elevated (993 nM), the decay of cytosolic Ca^2+^ upon removal of external Ca^2+^ was much slower (Rest [Ca^2+^] 0.53 ± 0.26 μM) and the Ca^2+^ release transient decayed faster. Peak Ca^2+^ release moderately increased (Peak [Ca^2+^] 3.71 ± 0.53 μM) but this α-syn-induced effect was clearly less pronounced than in wild-type LRB906 cells. Western blot analysis further indicated that α-syn was expressed at similar levels in LRB906 and *sec4*^*ts*^ cells (Figure 2D). These results therefore indicate that the mechanisms underlying the aberrant Ca^2+^ handling in α-syn overexpressing cells are only partially abrogated by Sec4 suppression.

As a next step, we also examined [Ca^2+^]_*in*_ and growth in wild-type LRB906 cells overexpressing *SEC4* (LRB906-pSEC4). As shown in Figure 2B, LRB906-pSEC4 cells displayed a similar intracellular Ca^2+^ pattern as WT cells but the peak level of the biphasic Ca^2+^ release was slightly higher. Also, the Ca^2+^ profile in LRB906-pSEC4 cells overexpressing α-syn (at similar levels as control cells – Figure 2D), remained similar to that seen in α-syn overexpressing wild-type cells though the decay of [Ca^2+^]_*in*_ after removal of extracellular Ca^2+^ was somewhat faster (Rest [Ca^2+^] 0.15 ± 0.23 μM) while the peak level of the monophasic Ca^2+^ release was higher (Peak [Ca^2+^] 2.86 ± 0.63 μM) (Table 1). Surprisingly, the overexpression of *SEC4* allowed the partial rescue of α-syn-induced growth inhibition (Figure 2C and Table 2).

**FIGURE 3:**
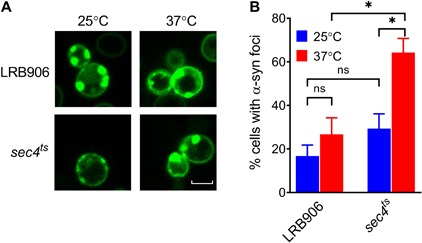
α-Syn aggregation in LRB906 and *sec4*^*ts*^ cells. **(A)** Confocal images of LRB906 and *sec4*^*ts*^ cells expressing α-syn-yeGFP prior to (25°C) and following incubation at restrictive temperature of 37°C for 1 h 15 min. Scale bar corresponds to 5 μm. **(B)** Bar graph showing percentage of cells containing α-syn foci (expressed as mean ± SD) under these experimental conditions. The percentage of cells with foci was determined by visual inspection of at least 300 cells. Results shown are representative of at least 3 independent experiments. The statistical significance was assessed using one-way ANOVA with Bonferroni posttest. ns (not significant) *P* > 0.05; *P ≤ 0.05.

To investigate whether aggregation of α-syn is required to induce [Ca^2+^]_*in*_ changes, we monitored aggregation in LRB906 and *sec4*^*ts*^ cells expressing α-syn fused with yeast-enhanced green fluorescent protein (yeGFP). Live-cell imaging of α-syn-yeGFP under these conditions revealed a significant percentage of cells displaying foci in both LRB906 (16.7 ± 8.8%) and *sec4*^*ts*^ cells at permissive temperatures (29.3 ± 11.8%). Upon shifting to restrictive temperatures the percentage of cells displaying foci greatly increased in both LRB906 (26.7 ± 13.1%) and *sec4*^*ts*^ cells (64.2 ± 11.2%), which in the latter was much more pronounced (Figure 3). We also noted that the percentage of cells that formed α-syn foci was not affected by overexpression of *SEC4* (Supplementary Figure S3).

### α-Syn Impairs Ca^2+^ Storage

**FIGURE 4:**
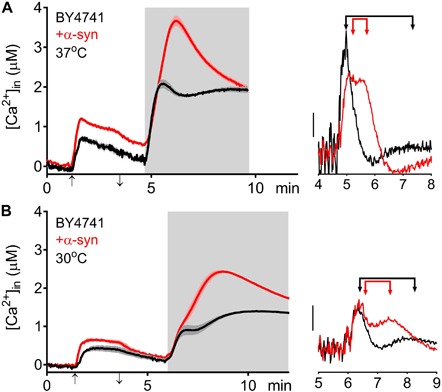
α-Syn overexpression in BY4741 cells. Averaged Ca^2+^ transient ± SEM (thin lines either side of the Ca^2+^ transient trajectory) of BY4741 (black traces) and BY4741 overexpressing α-syn (+α-syn – red traces) at 37 **(A)** and 30°C **(B)**. Cells were initially perfused with Ca^2+^-free starvation medium and then transferred to a 10 mM external Ca^2+^ medium for 2 min (indicated by up and down arrow on *X*-axis). Thereafter, cells were briefly exposed to Ca^2+^-free intracellular medium prior to membrane permeabilization using Triton X-100 (indicated by light gray zone). *Right insets*: first derivative of the Ca^2+^ release transients versus time showing fast and slow release components (indicated by arrowed bars). Scale bar corresponds to 1 μM/min.

**FIGURE 5:**
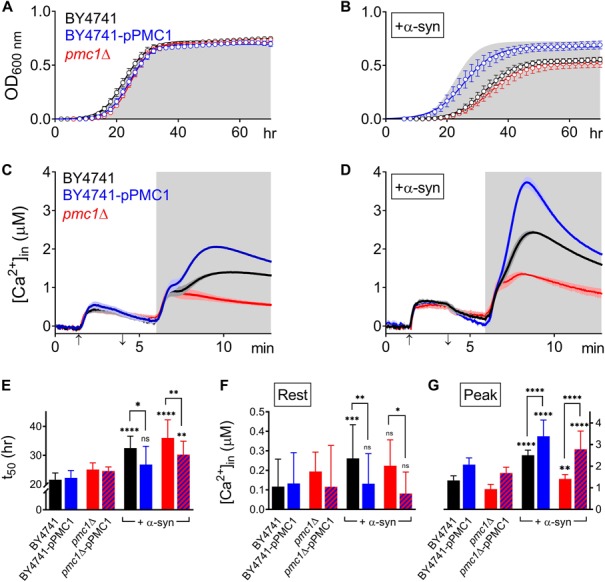
α-Syn overexpression in *PMC1* mutant strains. **(A,B)** Cell growth analysis of BY4741 yeast strains. OD_600_ measurements were obtained for BY4741 cells (black), cells overexpressing *PMC1* (BY4741-pPMC1 – blue) and *pmc1Δ* cells (red) without **(A)** or with overexpression of α-syn **(B)**. Gray shaded area in **(B)** corresponds to growth curve of control BY4741 cells **(A)**. Each time point represents the average ± SEM of triplicate cultures. **(C,D)** Averaged Ca^2+^ transient ± SEM (thin lines either side of the Ca^2+^ transient trajectory) of BY4741 cells (black), cells overexpressing *PMC1* (BY4741-pPMC1 - blue) and *pmc1Δ* cells (red) without **(C)** or with overexpression of α-syn **(D)** at 30°C. Cells were initially perfused with Ca^2+^-free starvation medium and then transferred to a 10 mM external Ca^2+^ medium for 2 min (indicated by up and down arrow on *X*-axis). Thereafter, cells were briefly exposed to Ca^2+^-free intracellular medium prior to membrane permeabilization using Triton X-100 (indicated by light gray zone). **(E–G)** Comparison of t_50_ (time to reach half-maximal OD_600_) **(C)**, Rest [Ca^2+^] ([Ca^2+^]_*in*_ value determined at the end of the Ca^2+^-free period prior to permeabilization) **(D)** and Peak [Ca^2+^] (peak amplitude of the Ca^2+^ release transient following permeabilization) **(E)** for *PMC1* mutant strains without and with α-syn overexpression. Bar graphs depicting mean ± SD. The statistical significance was assessed using one-way ANOVA with Bonferroni posttest. Unless otherwise indicated by connecting bar, differences were tested with respect to the control strain (without α-syn expression). ns (not significant) *P* > 0.05; *P ≤ 0.05; **P ≤ 0.01; ***P ≤ 0.001; ****P < 0.0001. Mean and *P*-values are listed in Tables 4, 5.

The higher [Ca^2+^]_*in*_ in 10 mM external Ca^2+^, the incomplete decay of cytosolic [Ca^2+^] in Ca^2+^ free medium and the faster decay of the Ca^2+^ release transient observed in wild-type and *sec4*^*ts*^ cells overexpressing α-syn clearly suggest that α-syn impairs cytosolic Ca^2+^ clearance and organellar Ca^2+^ storage. Since the vacuole is the main Ca^2+^ storage in yeast, we studied the effects of α-syn in strains lacking the vacuolar Ca^2+^ transporters Pmc1 and Vcx1 in the BY4741 background. To allow comparison with results obtained in wild-type LRB906 and *sec4*^*ts*^ strains at restrictive temperatures, we first checked Ca^2+^ responses in wild-type BY4741 cells at 37 and 30°C (Figure 4 and Table 3). Cooling (37 to 30°C) significantly decreased the rate of [Ca^2+^]_*in*_ changes but the overall effects of α-syn were still apparent including a higher resting [Ca^2+^]_*in*_ in 10 mM external Ca^2+^, an incomplete decay of cytosolic [Ca^2+^] in Ca^2+^ free medium, a shift from a biphasic to a quasi-monophasic Ca^2+^ release and the faster decay of the Ca^2+^ release transient. As shown by the first derivative of the Ca^2+^ release transient (Figure 4 – insets), it becomes also apparent that the α-syn-induced shift from a biphasic to a quasi-monophasic Ca^2+^ release mainly reflects an increase in both the rate of Ca^2+^ release and the amplitude of the second slow component, while the first fast component is minimally or not affected. In addition, the α-syn-induced growth inhibition observed in the BY4741 strain (Figures 5A,B,E and Table 4) was similar to that in LRB906 cells (Δt_50_ values of 12.3 and 10.9 h in LRB906 and BY4741, respectively). As 30°C is the optimum temperature for yeast and to minimize temperature stress, further experimental assays in the BY4741 background were performed at 30°C. Furthermore, as shown in Supplementary Figure S4, extracellular [Ca^2+^] is not an absolute requirement for the α-syn-induced shift from biphasic to quasi-monophasic Ca^2+^ release. When cells were not challenged with extracellular Ca^2+^, membrane permeabilization evoked a similar biphasic Ca^2+^ release as in cells challenged with 10 mM external [Ca^2+^]. Likewise, the response in α-syn overexpressing cells became quasi-monophasic although peak [Ca^2+^] was significantly reduced compared to cells previously exposed to external [Ca^2+^].

**TABLE 3:** [Ca^2+^]_*in*_ determined at the end of the Ca^2+^-free period (Rest [Ca^2+^]) (before membrane permeabilization) and the peak amplitude of the Ca^2+^ release transient (Peak [Ca^2+^]) in BY4741 cells and BY4741 cells overexpressing α-syn at 30 and 37°C.

	**BY4741**	**BY4741**
2*Rest [Ca^2+^] (μM)	**30°** 0.12 ± 0.14	**37°** 0.25 ± 0.30
	*n* = 41	*n* = 17
**BY4741+α-syn**		
**30°**	**	
0.26 ± 0.17	0.0088	
*n* = 25		
**BY4741+α-syn**		
**37°** 0.53 ± 0.20		**** <0.0001
*n* = 24		

	**BY4741**	**BY4741**
2*Peak [Ca^2+^] (μM)	**30°** 1.34 ± 0.21	**37°** 2.30 ± 0.44
	*n* = 41	*n* = 17

**BY4741+α-syn**		
**30°**	****	
2.51 ± 0.23	<0.0001	
*n* = 25		
**BY4741+α-syn**		
**37°** 4.25 ± 0.92		**** <0.0001
*n* = 24		

Data are mean and SD for n cultures (coverslips). Statistical differences were calculated using one-way ANOVA with Bonferroni posttest. **P ≤ 0.01; ****P < 0.0001.

**TABLE 4:** Growth of BY4741 yeast strains.

**Growth *t*_50_ (hr)**	**BY4741** 21.60 ± 2.35 *n* = 13	**BY4741-pPMCl** 22.25 ± 2.52 *n* = 13	***pmc1**Δ***** 25.12 ± 2.29 *n* = 14	***pmc1***Δ**-pPMC1** 24.60 ± 1.40 *n* = 15	***vcx1**Δ***** 24.71 ± 1.70 *n* = 6	***vcx1**Δ***-pPMC1** 24.43 ± 1.13 *n* = 6	**BY4741+α-syn** 32.52 ± 4.11 *n* = 13	***pmc1**Δ***+α-syn** 36.00 ± 6.25 *n* = 13	***vcx1**Δ***+α-syn** 38.32 ± 8 03 *n* = 9
**BY4741+α-syn** 32.52 ± 4.11 *n* = 13	**** <0.0001								
**BY4741-pPMC1+α-syn** 26.S3 ± 6.26 *n* = 12		ns 0.1002					* 0.0195		
***pmc1Δ*+α-syn** 36.00 ± 6.25 *n* = 13			**** <0.0001						
***pmc1Δ*-pPMC1+α-syn** 30.25 ± 4.61 *n* = 15				** 0.0014				** 0.0018	
***vcx1Δ+α-**syn***** 38.32 ± 8.03 *n* = 9					**** <0.0001				
***vcx1Δ*-pPMC1+α-syn** 36.90 ± 5.81 *n* = 10						**** <0.0001			ns >0.0999

Equal number of cells were grown at 25°C. *t*_50_ values are mean and SD for at least 6 replicates. Statistical differences were calculated using one-way ANOVA with Bonferroni posttest. ns (not significant) *P* > 0.05; *P ≤ 0.05; **P ≤ 0.01; ****P < 0.0001.

**TABLE 5:** [Ca^2+^]_*in*_ determined at the end of the Ca^2+^-free period (Rest [Ca^2+^]) (before membrane permeabilization) and the peak amplitude of the Ca^2+^ release transient (Peak [Ca^2+^]) in BY4741 yeast strains at 30°C.

**Rest [Ca^2+^] (μM)**	**BY4741** 0.12 ± 0.14 *n* = 41	**BY4741-pPMCl** 0.13 ± 0.16 *n* = 45	***pmc1**Δ***** 0.19 ± 0.10 *n* = 25	***pmc1**Δ**-*pPMC1** 0.12 ± 0.21 *n* = 24	***vcx1**Δ***** 0.05 ± 0.10 *n* = 26	***vcx1**Δ**-*pPMC1** 0.08 ± 0.12 *n* = 23	**BY4741+α-syn** 0.26 ± 0.17 *n* = 25	***pmc1**Δ** + α-**syn***** 0.22 ± 0.13 *n* = 17	***vcx1**Δ** + α-**syn***** 0.12 ± 0.15 *n* = 27
**BY4741+α-syn** 0.26 ± 0.17 *n* = 25	*** 0.0005								
**BY4741-pPMC1+α-syn** 0.13 ± 0.15 *n* = 31		ns >0.9999					* 0.0049		
***pmc1Δ+α-**syn***** 0.22 ± 0.13 *n* = 17			ns >0.9999						
***pmc1Δ-*pPMC1 + α-syn** 0.08 ± 0.11 *n* = 20				ns >0.9999				* 0.0256	
***vcx1Δ*+α-syn** 0.12 ± 0.15 *n* = 27					ns 0.4881				
***vcx1Δ-*pPMC1+α-syn** 0.06 ± 0.11 *n = 26*						ns >0.9999			ns 0.8780

**Peak [Ca^2+^] (μM)**	**BY4741** 1.34 ± 0.21 *n* = 41	**BY4741-pPMC1** 2.07 ± 0.31 *n* = 45	***pmc1***Δ**** 0.94 ± 0.22 *n* = 25	***pmc1**Δ***-pPMC1** 1.64 ± 0.27 *n* = 24	***vcx1**Δ***** 1.39 ± 0.11 *n* = 26	***vcx1**Δ***-pPMC1** 2.22 ± 0.37 *n* = 23	**BY4741+α-syn** 2.51 ± 0.23 *n* = 25	***pmc1**Δ***+α-**syn**** 1.41 ± 0.20 *n* = 17	***vcx1**Δ***+α-**syn**** 2.25 ± 0.15 *n* = 27

**BY4741+α-syn** 2.51 ± 0.23 *n* = 25	**** <0.0001								
**BY4741-pPMC1+α-syn** 3.38 ± 0.75 *n* = 31		**** <0.0001					**** <0.0001		
***pmc1Δ*+α-syn** 1.41 ± 0.20 *n* = 17			** 0.0038						
***pmc1Δ*-pPMC1+α-syn** 2.78 ± 0.84 *n* = 20				**** <0.0001				**** <0.0001	
***vcx1*Δ+α-syn** 2.25 ± 0.15 *n* = 27					**** <0.0001				
***vcx1Δ-*pPMC1+α-syn** 3.59 ± 0.27 *n* = 26						**** <0.0001			**** <0.0001

Data are mean and SD for n cultures (coverslips). Statistical differences were calculated using one-way ANOVA with Bonferroni posttest. ns (not significant) *P* > 0.05; *P ≤ 0.05; **P ≤ 0.01; ***P ≤ 0.001; ****P< 0.0001.

For the BY4741 strains, the [Ca^2+^]_*in*_ values determined at the end of the Ca^2+^-free period before permeabilization (Rest [Ca^2+^]) as well as the peak amplitude of the Ca^2+^ release transient taken as the maximum of the biphasic Ca^2+^ release transient (Peak [Ca^2+^]) are listed in Table 5.

Pmc1 is the high affinity/low capacity vacuolar Ca^2+^ transporter in yeast cells that mainly controls resting [Ca^2+^]_*in*_ (D’hooge et al., 2015). As compared to wild-type BY4741 cells (Figure 5C – black trace), *pmc1Δ* cells displayed an increase in resting [Ca^2+^]_*in*_ value (Figure 5C – red trace; Figure 5F) and a small Ca^2+^ release transient upon membrane permeabilization, consisting of the fast Ca^2+^ release component (Figure 5C – red trace; Figure 5G). Upon overexpression of α-syn in *pmc1Δ* cells, resting [Ca^2+^]_*in*_ slightly enhanced while a small second component appeared in the Ca^2+^ release transient (Figure 5D – red trace; Figures 5F,G). BY4741 cells overexpressing *PMC1* (BY4741-pPMC1) showed similar resting [Ca^2+^]_*in*_ value as wild-type BY4741 cells (Figure 5C – blue trace; Figure 5F) but an enhanced biphasic release transient (Figure 5C – blue trace; Figure 5G). Overexpression of α-syn in the BY4741-pPMC1 strain resulted in a large quasi-monophasic Ca^2+^ release transient but resting [Ca^2+^] was not affected (Figure 5D – blue trace; Figures 5F,G). This result suggests that the α-syn-induced shift from a biphasic to a quasi-monophasic Ca^2+^ release may reflect rapid Ca^2+^ release form an additional pool of vesicles loaded with Ca^2+^ by Pmc1. To further address this point, we studied Pmc1 localization in FM4-64 stained yeast cells. As evident from Supplementary Figure S5, BY4741 control cells typically showed one large vacuole containing Pmc1. By contrast, in BY4741 cells expressing α-syn Pmc1 was found in numerous small vesicle-like structures and FM4-64 staining identified these structures as vacuolar fragments.

Finally, overexpression of *PMC*1 rescued α-syn induced toxicity on growth both in wild-type (BY4741-pPMC1) and *pmc1Δ* (*pmc1Δ*-pPMC1) cells (Figure 5B,E and Table 4).

**FIGURE 6:**
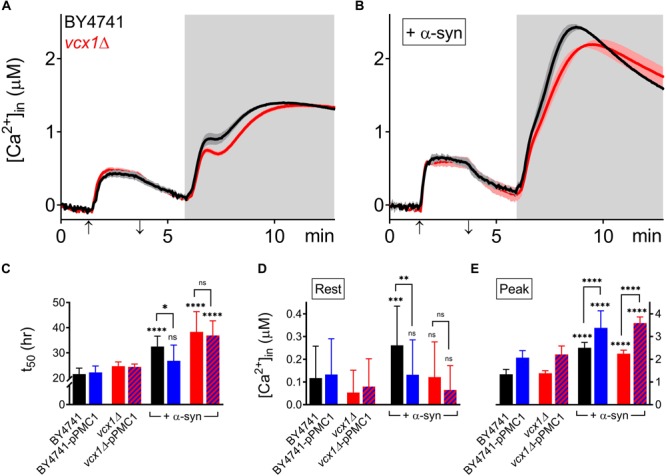
α-Syn overexpression in *VCX1* mutant strains. **(A,B)** Averaged Ca^2+^ transient ± SEM (thin lines either side of the Ca^2+^ transient trajectory) of BY4741 cells (black) and *vcx1Δ* cells (red) without **(A)** or with overexpression of α-syn **(B)** at 30°C. Cells were initially perfused with Ca^2+^-free starvation medium and then transferred to a 10 mM external Ca^2+^ medium for 2 min (indicated by up and down arrow on *X*-axis). Thereafter, cells were briefly exposed to Ca^2+^-free intracellular medium prior to membrane permeabilization using Triton X-100 (indicated by light gray zone). **(C–E)** Comparison of *t*_50_ (time to reach half-maximal OD_600_) **(C)**, Rest [Ca^2+^] ([Ca^2+^]_*in*_ value determined at the end of the Ca^2+^-free period prior to permeabilization) **(D)** and Peak [Ca^2+^] (peak amplitude of the Ca^2+^ release transient following permeabilization) **(E)** for *VCX1* mutant strains without and with α-syn overexpression. Bar graphs depicting mean ± SD. The statistical significance was assessed using one-way ANOVA with Bonferroni posttest. Unless otherwise indicated by connecting bar, differences were tested with respect to the control strain (without α-syn expression). ns (not significant) *P* > 0.05; ^∗^*P* ≤ 0.05; ^∗∗^*P* ≤ 0.01; ^∗∗∗^*P* ≤ 0.001; ^****^P < 0.0001. Mean and *P*-values are listed in Tables 4, 5.

**FIGURE 7:**
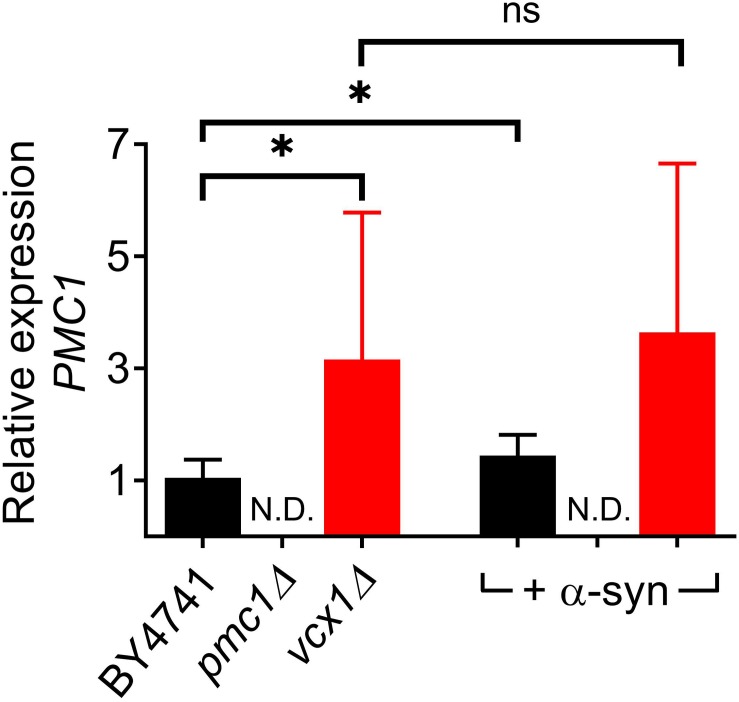
Relative *PMC1* expression levels in *vcx1Δ* strains. Bar graph depicting relative mRNA levels of *PMC1* in BY4741, *pmc1Δ* and *vcx1Δ* cells and cells overexpressing α-syn. Bars represent the means ± SD of at least 6 independent quantitative PCR assays. The statistical significance was assessed using unpaired Student’s *t*-test. ns (not significant) *P* > 0.05; *P ≤ 0.05; N.D, non-detectable.

The *vcx1Δ* strain was also tested (Figure 6). Vcx1 has been characterized as a low affinity/high capacity vacuolar Ca^2+^/H^+^ antiporter in yeast cells (Cagnac et al., 2010). Compared to wild-type BY4741 cells, *vcx1Δ* cells displayed significantly decreased [Ca^2+^]_*in*_ values in Ca^2+^-free medium (Rest [Ca^2+^] 0.05 ± 0.10 μM – Table 5), but the Ca^2+^ release transient was minimally affected and remained biphasic (Figure 6A – red trace; Figures 6D,E). Remarkably, overexpression of α-syn in *vcx1Δ* cells did not significantly increase [Ca^2+^]_*in*_ in Ca^2+^ free medium (Rest [Ca^2+^] 0.12 ± 0.15 μM) but the Ca^2+^ release transient became nearly monophasic with a significantly higher peak value (Figure 6B – red trace; Figures 6D,E). *vcx1Δ* cells overexpressing *PMC1* (*vcx1Δ*-pPMC1) showed similar resting [Ca^2+^]_*in*_ as *vcx1Δ* cells but an enhanced biphasic release transient (Figures 6D,E). Overexpression of α-syn in the *vcx1Δ*-pPMC1 strain resulted in a larger Ca^2+^ release transient but resting [Ca^2+^] and growth rate were not significantly affected (Figures 6C–E). To further investigate this phenotype, we also measured *PMC1* expression using qPCR (Figure 7). Wild-type BY4741 cells expressing α-syn showed a slight yet significant increase in *PMC1* expression. However, a stronger *PMC1* expression was observed in both *vcx1Δ* and *vcx1Δ +*α-syn cells.

## Discussion

In this work, we particularly focused on the effects of α-syn on yeast Ca^2+^ homeostasis. Overexpression of α-syn in wild-type yeast cells affects Ca^2+^ clearance (as indicated by incomplete Ca^2+^ decay upon removal of extracellular Ca^2+^, denoted as the Rest [Ca^2+^]) and storage (as indicated by a shift from a biphasic to a quasi-monophasic Ca^2+^ release transient upon membrane permeabilization, denoted as the Peak [Ca^2+^]).

Yeast cells overexpressing α-syn showed increased cytosolic Ca^2+^ levels in Ca^2+^ free medium (increased Rest [Ca^2+^]). This increase may result from an enhanced Ca^2+^ efflux from intracellular stores and/or a reduced organellar Ca^2+^ uptake. Since several studies have demonstrated that α-syn may form Ca^2+^ permeable pores (Quist et al., 2005; Schmidt et al., 2012; Stockl et al., 2013; Angelova et al., 2016), the increased cytosolic Ca^2+^ levels may simply reflect increased Ca^2+^ leakage from intracellular Ca^2+^ stores. Results obtained in *PMC1* overexpressing strains are in line with this conclusion. Indeed, the reduced effect of α-syn in *PMC1* overexpressing cells could be easily explained by increased Pmc1 Ca^2+^ pumping activity, which opposes the α-syn-induced leakage. Similarly, the reduced effect of α-syn on Rest [Ca^2+^] in *vcx1Δ* cells likely reflects the compensatory increased expression of *PMC1* in this deletion strain.

In wild-type yeast cells, a biphasic Ca^2+^ release transient was consistently found. In previous work (D’hooge et al., 2015), we have postulated that the initial fast phase mainly reflects free releasable Ca^2+^ from organelles (including ER, Golgi, mitochondria, vacuole and vesicle pools) while the second slower phase reflects the release of organellar bound Ca^2+^ (mainly Ca^2+^ bound to vacuolar polyphosphates). The finding that the deletion of Pmc1, the main vacuolar Ca^2+^ transporter, strongly reduces the second slower component further supports this hypothesis.

Yeast cells overexpressing α-syn typically showed a shift from biphasic to a quasi-monophasic release of stored Ca^2+^. Several studies have demonstrated that α-syn overexpression strongly inhibits vesicle trafficking (Cooper et al., 2006; Larsen et al., 2006; Gitler et al., 2008; Lee et al., 2011; Sancenon et al., 2012; Wang and Hay, 2015). To investigate whether changes in vesicle trafficking may underlie the shift from a biphasic to a quasi-monophasic Ca^2+^ release, we used yeast cells carrying a temperature-sensitive (ts) mutation in *SEC4* (*sec4*^*ts*^). Our reigning hypothesis is that dysfunction of Sec4 disrupts exocytosis (Novick et al., 1993; Brennwald et al., 1994) and therefore significantly increases the size of the pool of vesicles. Assuming that vesicles are Ca^2+^ loaded, the number of vesicles available for rapid Ca^2+^ release in response to membrane permeabilization will affect the amplitude and kinetics of the Ca^2+^ release transient. As expected, at restrictive temperature *sec4*^*ts*^ cells displayed a fast-rising Ca^2+^ release transient which presumably reflects a fast Ca^2+^ release from stocked vesicles. Because a similar fast-rising Ca^2+^ release transient was observed in yeast cells overexpressing α-syn, we propose that the shift of biphasic to quasi-monophasic Ca^2+^ release transient in α-syn overexpressing cells may reflect an increase in the pool size of vesicles that are no longer able to undergo fusion. In α-syn overexpressing cells, the accumulation of Ca^2+^ into these vesicles seems to be dependent on Pmc1 activity, as based on the changes in Peak [Ca^2+^] in parallel with *PMC1* expression levels (compare *pmc1Δ*, BY4741 and BY4741-pPMC1 cells). Furthermore, while control cells showed a typical single large Pmc1-containing vacuole, cells expressing α-syn accumulated multiple small Pmc1-containing vesicles. Co-staining with endocytic marker FM4-64 revealed co-localization with vacuolar membranes. In accordance with these observations, we therefore propose that the additional pool of vesicles constitute fragmentation and vesiculation of vacuolar membranes, which display fewer fusion events as a consequence of α-syn interaction with vesicle trafficking components (Gitler et al., 2008; Zabrocki et al., 2008; Soper et al., 2011; Bridi and Hirth, 2018; Huang et al., 2019). Additionally, increased organellar Ca^2+^ leakage (α-syn pore forming activity) is expected to shorten the duration of the Ca^2+^ release transient and to reduce the time to peak [Ca^2+^] release.

The decay phase of the Ca^2+^ release transient was faster in α-syn expressing cells. In permeabilized cells, the rate of decay of the Ca^2+^ release transient reflects a balance between organellar Ca^2+^ release and Ca^2+^ leakage across the plasma membrane. In α-syn overexpressing cells, the Ca^2+^ release occurs at high cytosolic Ca^2+^ levels (peak [Ca^2+^] 2.51 ± 0.23 μM in BY4741 +α-syn) while this occurs in control cells at much lower levels (peak [Ca^2+^] 1.34 ± 0.21 μM in BY4741). Therefore, the initial Ca^2+^ leak across the plasma membrane will be significantly larger in the BY4741 +α-syn cells resulting in a faster decay of the Ca^2+^ release component. Additionally, as organellar Ca^2+^ leakage is likely to be increased (α-syn pore forming activity) in α-syn overexpressing cells, the total Ca^2+^ vacuolar storage will be lower resulting in a faster decay of the Ca^2+^ release component.

Finally, overexpression of *PMC1* in BY4741 and in *pmc1Δ* abolished the α-syn-related cytotoxicity on growth. As Peak [Ca^2+^] increased and Rest [Ca^2+^] decreased in parallel with *PMC1* expression levels, this observation suggests that the cytotoxicity of α-syn is closer related to its propensity to form pores rather than disrupting vesicle trafficking.

However, important to note is that overexpression of *PMC1* in *vcx1Δ* cells did not reduce α-syn-induced toxicity. In *vcx1Δ* cells, it was found that *PMC1* expression was enhanced. As *PMC1* is a calcineurin-dependent gene and calcineurin may contribute to α-syn-induced toxicity (Caraveo et al., 2014) these findings raise the possibility that loss of Vcx1 function or calcineurin activity may create a cellular environment that augments α-syn-induced toxicity and warrants further investigation.

## Data Availability Statement

All datasets generated for this study are included in the article/Supplementary Material.

## Author Contributions

GC and JW supervised the project. MD, VV, T-YM, and PD’h performed the experiments and analyzed the data. VF provided yeast strains and vectors. VV, T-YM, and GC prepared the manuscript. All authors revised the manuscript.

## Conflict of Interest

JW declares that he is co-founder and shareholder of the KU Leuven spin-off companies reMYND nv (Leuven, Belgium) and ADx NeuroSciences nv (Ghent, Belgium), but this did not influence in any way the studies reported in the manuscript.

The remaining authors declare that the research was conducted in the absence of any commercial or financial relationships that could be construed as a potential conflict of interest.
