# Supplementary material for: Decreased Vacuolar Ca2+ Storage and Disrupted Vesicle Trafficking Underlie Alpha-Synuclein-Induced Ca2+ Dysregulation in S. cerevisiae
Source: Front Genet. 2020 May 8;11:266. doi: 10.3389/fgene.2020.00266 (PMC7225347; doi:10.3389/fgene.2020.00266)
Supplement: Supplementary file 1 [file Data_Sheet_1.docx]

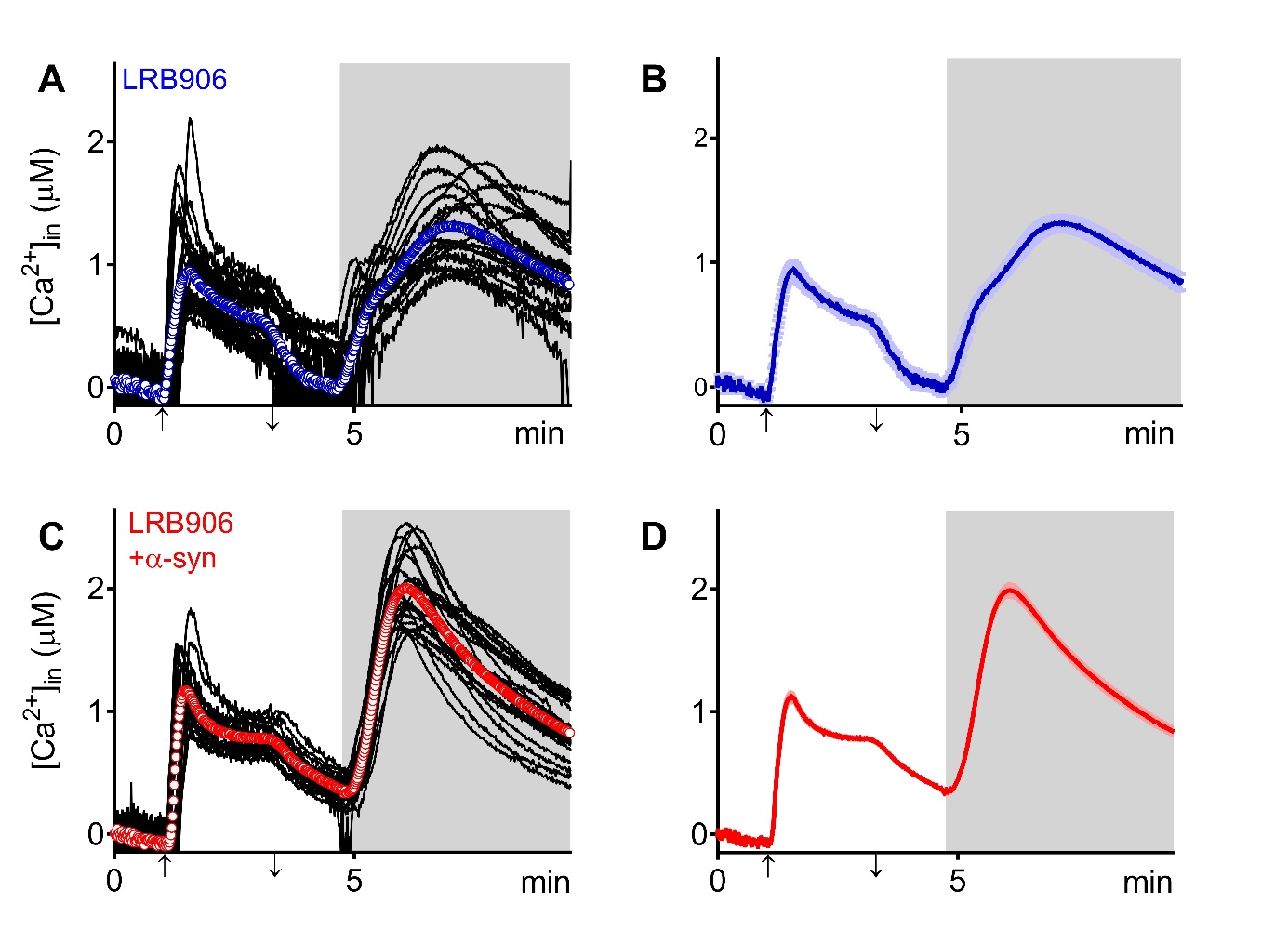


**Supplemental Figure 1**. Ca^2+^ signal responses from yeast cell populations grown on concanavaline A-coated glass coverslips (black traces) and ensemble average of Ca^2+^ values ± SEM (shaded areas either side of the Ca^2+^ signal trajectory) of LRB906 cells (blue trace in **A** and **B**) and LRB906 cells overexpressing α-syn (red trace in **C** and **D**) at 37°C. Cells were initially perfused with Ca^2+^-free starvation medium and then transferred to a 10 mM external Ca^2+^ medium for 2 min (indicated by up and down arrow on X-axis). Thereafter, cells were briefly exposed to Ca^2+^-free intracellular medium prior to membrane permeabilization using Triton X-100 (indicated by light grey zone).


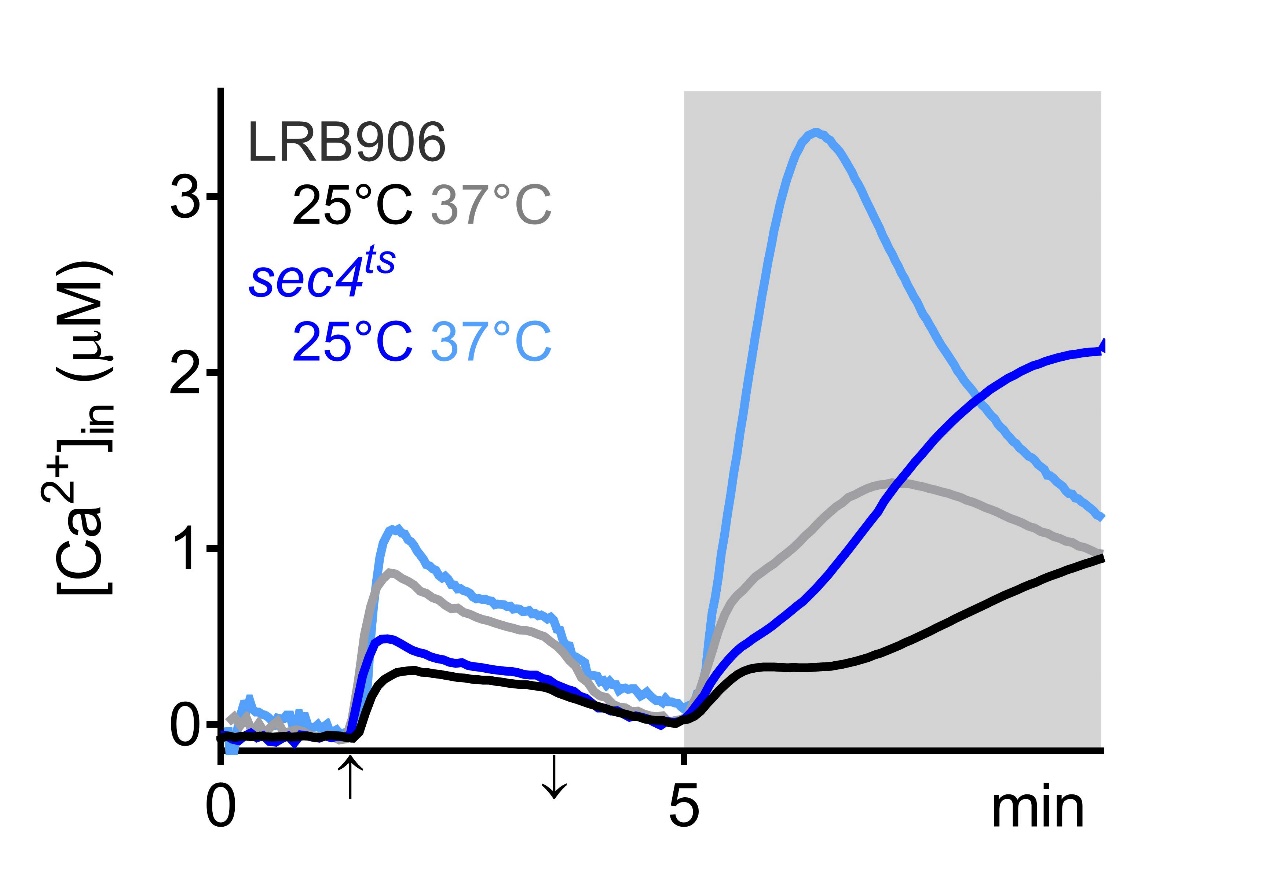


**Supplemental Figure 2**. Averaged Ca^2+^ transient ± SEM (thin lines either side of the Ca^2+^ transient trajectory) of LRB906 and *sec4^ts^* cells at 25 and 37°C. Cells were initially perfused with Ca^2+^-free starvation medium and then transferred to a 10 mM external Ca^2+^ medium for 2 min (indicated by up and down arrow on X-axis). Thereafter, cells were briefly exposed to Ca^2+^-free intracellular medium prior to membrane permeabilization using Triton X-100 (indicated by light grey zone).

**
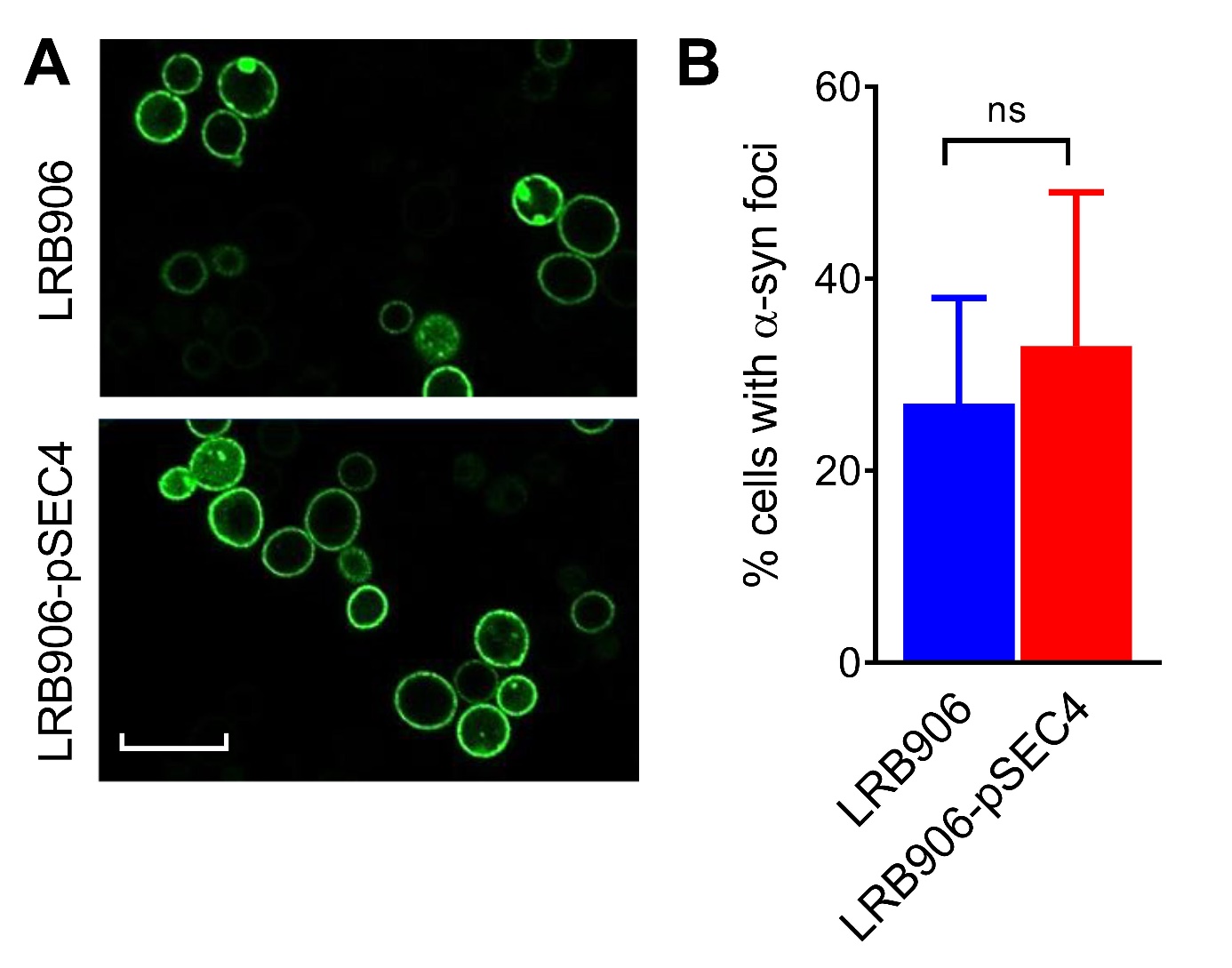
**

**Supplemental Figure 3**. α-Syn aggregation in LRB906 and LRB906-pSEC4 cells. (**A**). Confocal images of LRB906 and LRB906-pSEC4 cells overexpressing α-syn-yeGFP following incubation at 37°C for 1 h 15min. Scale bar corresponds to 10 μm. (**B**). Bar graph showing percentage of cells containing α-syn foci (expressed as mean ± SD) under these experimental conditions. The percentage of cells with foci was determined by visual inspection of at least 1000 cells. Results shown are representative of at least 3 independent experiments. The statistical significance was assessed using unpaired Student’s t-test (ns (not significant) P=0.1).

**
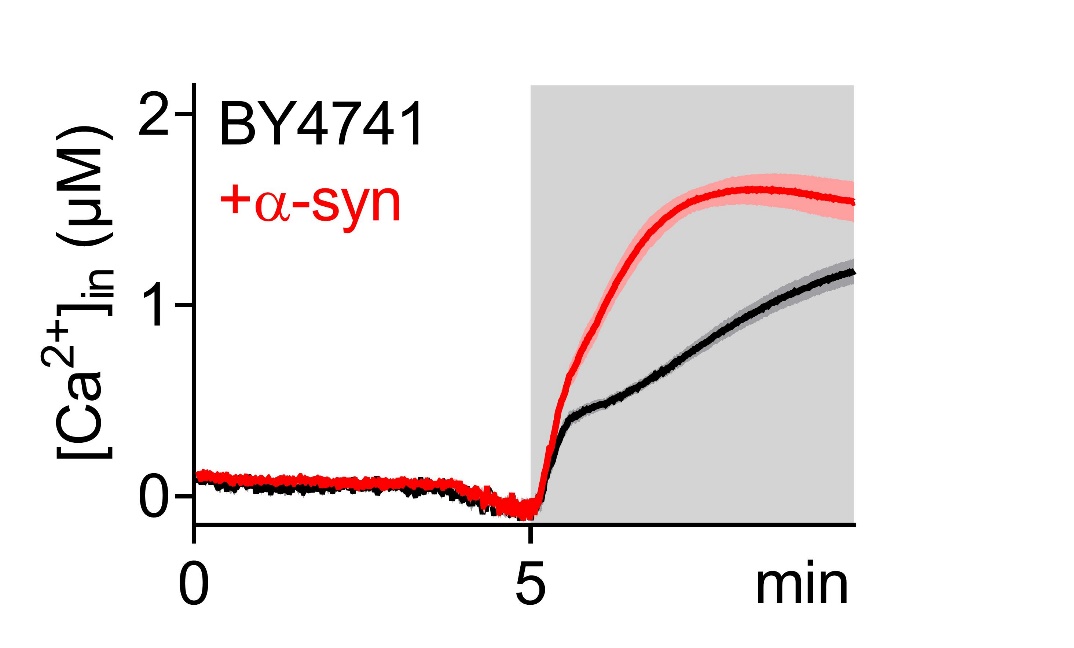
**

**Supplemental Figure 4**. Averaged Ca^2+^ transient ± SEM (thin lines either side of the Ca^2+^ transient trajectory) of BY4741 (black trace) and BY4741 cells overexpressing α-syn (+α-syn - red trace) at 30°C. Cells were perfused with Ca^2+^-free starvation medium and then briefly exposed to Ca^2+^-free intracellular medium prior to membrane permeabilization using Triton X-100 (indicated by light grey zone). Peak [Ca^2+^] in α-syn expressings cells was 1.78±0.21 μM (n=8) compared to 2.00±0.16 μM (n=7) obtained in cells challenged with 10 mM external Ca^2+^ prior to permeabilization (* P ≤ 0.05, determined using unpaired Student’s t-test).


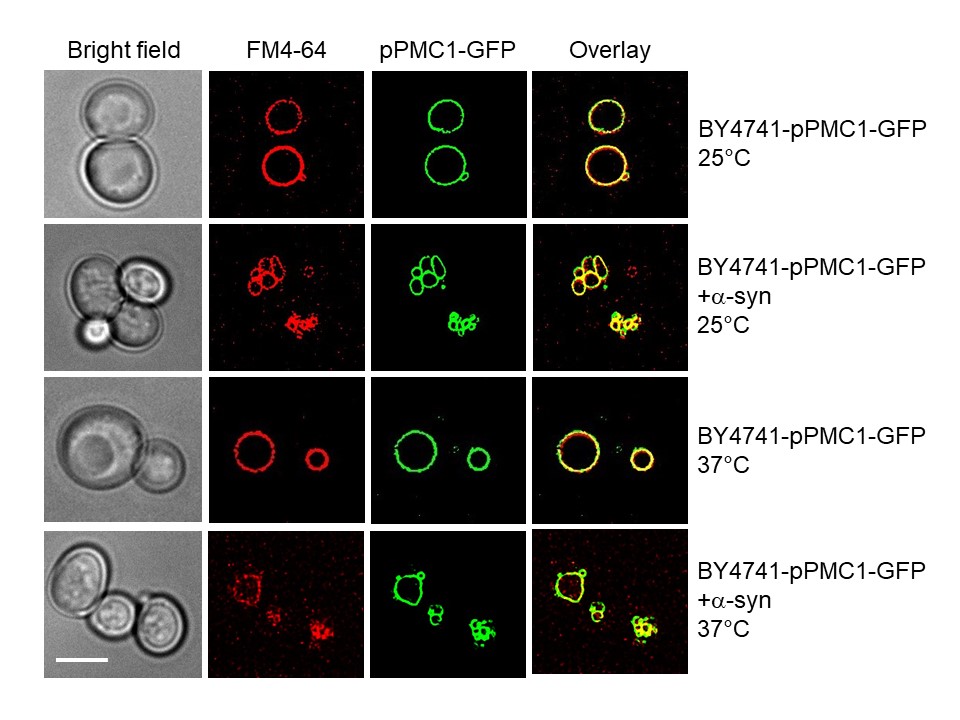


**Supplemental Figure 5**. Effects of α-syn expression on Pmc1 distribution and vacuolar morphology in BY4741 cells at 25 and 37°C. To visualize Pmc1 distribution in α-syn expressing cells, BY4741 cells were co-transformed with pPMC1-GFP fusion plasmid and pGGE181-α-syn. Cells co-transformed with pGGE181-EV served as control. FM4-64 staining (red) was used to identify vacuoles and corroborate that Pmc1–GFP (green) localized to vacuole membranes (overlay – colocalization shown in yellow). Scale bar corresponds to 5 μm.
